# Specific CD8^+^ TCR Repertoire Recognizing Conserved Antigens of SARS-CoV-2 in Unexposed Population: A Prerequisite for Broad-Spectrum CD8^+^ T Cell Immunity

**DOI:** 10.3390/vaccines9101093

**Published:** 2021-09-28

**Authors:** Wei Hu, Meifang He, Xiaoning Wang, Qiang Sun, Ming Kuang

**Affiliations:** 1 Department of Oncology, The First Affiliated Hospital of Sun Yat-sen University, Guangzhou 510080, China; huwei57@mail.sysu.edu.cn; 2 Laboratory of General Surgery, The First Affiliated Hospital of Sun Yat-sen University, Guangzhou 510080, China; hemeifang@mail.sysu.edu.cn; 3 National Clinical Research Center for Geriatrics Diseases, Chinese PLA General Hospital, Beijing 100853, China; xiaoningwang@cuc.edu.cn; 4 School of Laboratory Medicine and Biotechnology, Southern Medical University, Guangzhou 510515, China; 5 Laboratory of Cell Engineering, Institute of Biotechnology, Beijing 100071, China; sunq@bmi.ac.cn; 6 Research Unit of Cell Death Mechanism, 2020RU009, Chinese Academy of Medical Science, Beijing 100071, China; 7 Division of Interventional Ultrasound, The First Affiliated Hospital of Sun Yat-sen University, Guangzhou 510080, China; 8 Department of Liver Surgery, The First Affiliated Hospital of Sun Yat-sen University, Guangzhou 510080, China; 9 Cancer Center, The First Affiliated Hospital of Sun Yat-sen University, Guangzhou 510080, China

**Keywords:** SARS-CoV-2, broad-spectrum adaptive immunity, CD8^+^ T cell, TCR repertoire, vaccine

## Abstract

Background: Severe acute respiratory syndrome coronavirus 2 (SARS-CoV-2) has developed variants escaping neutralization antibody immunity established against the original virus. An understanding of broad-spectrum adaptive immunity, including CD8^+^ T cell immunity to wide range of epitopes, could help translational efforts to improve coronavirus disease 2019 (COVID-19) prevention and therapy. However, there have been few direct studies in which such immunity exists in a population. Methods: We selected SARS-CoV-2-conserved structural peptides that are not prone to mutation as antigens for broad-spectrum CD8^+^ T cell immunity. Peripheral blood mononuclear cells (PBMCs) from unexposed healthy donors were stimulated with these peptides in vitro and CD8^+^ T cell-specific response was monitored. The conserved peptide-specific CD8^+^ T cells were sorted for T cell receptor (TCR) repertoire sequencing. The presence of specific complementary determining region 3 (CDR3) clones was analyzed in a healthy cohort. Results: For each structural protein, including S, E, M, N, the conserved peptides could potentially provide the largest number of major histocompatibility complex-I (MHC-I) epitopes in the Oriental and Caucasian populations. For conserved peptides from spike (S), envelope (E), membrane (M), nucleocapsid (N) proteins, we found that there were no cross-reactive memory T cells in the unexposed individuals. Instead, their T cells contain naïve TCR repertoire recognizing these conserved peptides. Using TCR sequencing and CDR3 clustering for the conserved peptides specific T cells, we found that the recovered patients had a higher proportion of TCR repertoire similar with that of specific CD8^+^ T cells in unexposed individuals. Meanwhile, CDR3 clones of the above T cells were widely present in the healthy population. Conclusions: This study provides evidence of broad-spectrum SARS-CoV-2 specific CD8^+^ TCR repertoire in unexposed healthy population, which is implicated in the development and implementation of broad-spectrum vaccines against COVID-19.

## 1. Introduction

The pandemic of coronavirus disease 2019 (COVID-19), caused by the infection of the severe acute respiratory syndrome coronavirus 2 (SARS-CoV-2), has raged around the world for more than one year. Although available vaccines were promising in population protection, the virus kept evolving to produce a number of variants [1,2,3], with increasing variants turned out to be able to escape neutralization by natural and vaccine-induced sera [4,5]. The epidemic Delta variant started in India (B.1.617.2) and the rapidly expanding epidemic Lambda variant (B 1.1.1), first detected in Peru, underscores the seriousness of this problem [6,7]. The usual prevention strategy is developing new vaccines against these variants, as in the case of flu vaccine development [8]. However, the strategy is falling short of timely coping with the ever-emerging variants of concerns that display enhanced transmissibility [9,10]. This challenging public crisis calls for broad-spectrum SARS-CoV-2 immunity, which could be achieved by targeting conserved antigenic epitopes across SARS-CoV-2 strains.

The protective immunity of adaptive immune system consists of two arms, B cells for antibody immunity and T cell immunity including CD4^+^ T cells as the helper T cells, and CD8^+^ T cells as the cytotoxic cells. Neutralizing antibodies can prevent the binding and entry of virus into cell, which could prevent the initial implantation of virus at the portal of entry. However, this arm often faced with the problems of low titer, short duration, and immune escape of mutant [11,12,13,14]. As a supplement to antibodies, CD8^+^ T cells can effectively prevent the establishment of infection by clearing the infected cells in the initial few days after exposure [15], especially true of tissue-resident memory T cells (TRMs) [16]. However, CD4^+^ T cells cannot be considered to have the function of preventing the initial implantation of virus at the portal of entry. Instead, they tend to limit disease severity and duration of the disease [17,18]. Recent studies reported a robust and long-lasting T cell immunity in convalescent individuals with asymptomatic or mild COVID-19, of which CD8^+^ T cells played a major role in eliminating the infected cells [19,20].

Several reports have shown that pre-existing memory T cells that recognize SARS CoV-2 in a significant fraction of individuals not exposed to SARS-CoV-2 [21,22,23,24,25,26,27]. Interestingly, a recent publication indicated the presence of cross-reactive CD8^+^ memory T cells in the unexposed people, although at a much smaller rate than the CD4^+^ T cell response [28]. This pre-existing cross-reactive anti-SARS-CoV-2 T cell immunity in unexposed people, including CD4^+^ and CD8^+^, is thought to result from previous stimulation by other human coronaviruses (hCoVs). However, these studies did not address the issues of broad-spectrum SARS-CoV-2-specific CD8^+^ T cell immunity, which is tolerant to viral mutation. Conserved antigens may be an ideal immune target because it is not prone to mutate, and its specific T cell will have broad-spectrum characteristics in recognizing SARS-CoV-2 variants. In the current study, we determined whether CD8^+^ T cell receptor (TCR) repertoire recognizing the SARS-CoV-2 conserved antigens might exist in unexposed healthy people. This is fundamental for human to immune against SARS-CoV-2 and provides a new way for improving human immunity against mutant viruses.

## 2. Materials and Methods

### 2.1. Human Subjects

All donors were Oriental from the mainland of China, including unexposed healthy donors and recover patients. Donors were SARS-CoV-2 PCR test negative before recruitment. Healthy donors underwent epidemiological investigation to exclude those with potential exposure to SARS-CoV-2, while recovered patients provided SARS-CoV-2 antibody test results and proof of 2-weeks quarantine.

### 2.2. Epitope Prediction and Peptides Synthesis

**Figure 1:**
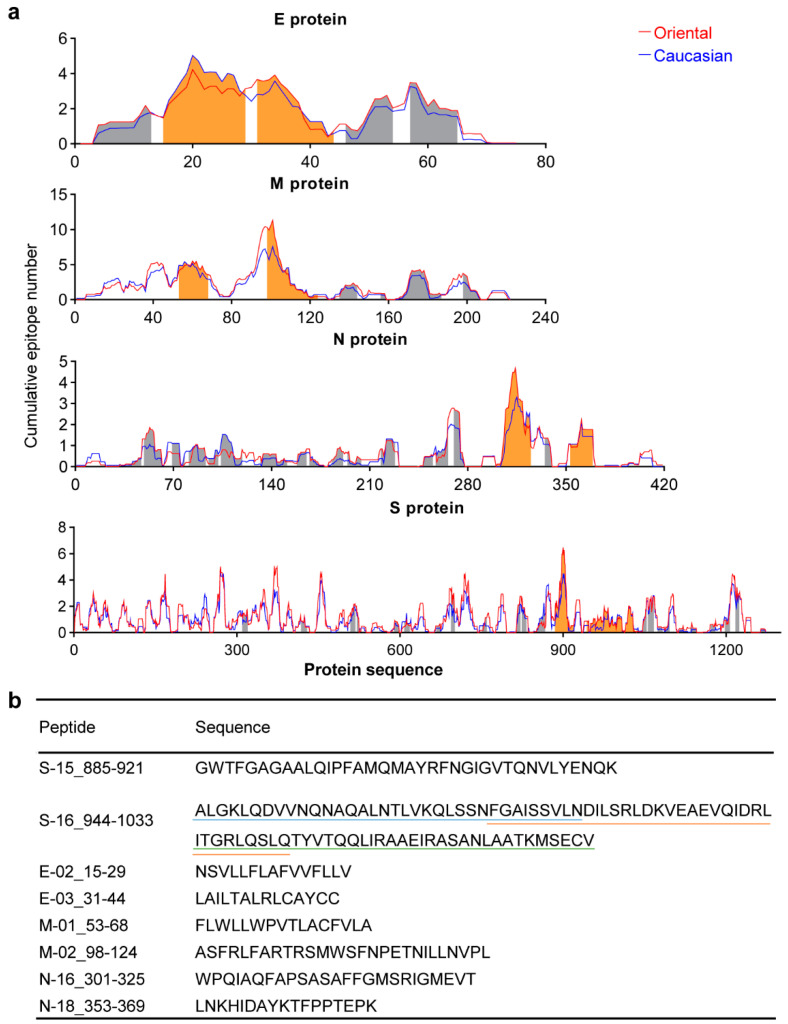
Selection of severe acute respiratory syndrome (SARS)-like viral conserved antigens. (**a**) the cumulative number of predicted major histocompatibility complex (MHC) class I epitopes in each amino acid of S, E, M, and N proteins for Oriental and Caucasian populations. NetMHCpan 4.0 was used for epitope prediction, and the number of epitopes was weighted for MHC alleles positive ratios. There was no significant difference between the two populations in the epitope curves of the four proteins. Shaded areas marked the conserved sequences among SARS-like viruses, of which the orange ones were selected as conserved antigens for the following study. (**b**) Sequences of the 8 selected conserved antigens in (**a**), with S16 being divided into three overlapping sequences. The synthesis of E2 and E3 failed because of the hydrophobicity. The combination of 8 synthetic peptides from S, M, N constituted the peptide pool.

In previous studies, we collected and calculated the major histocompatibility complex-I (MHC-I) allele positive ratios in Oriental and Caucasian populations [29]. Epitope binding affinity to MHC-I alleles of each population was predicted for SARS-CoV-2 structural proteins using NetMHCpan 4.0. The epitope length was restricted to 8–11 mer, and selected epitopes with affinity (nM) ≤ 200. Weighted the epitope number to the corresponding HLA allele positive ratio in each population, and then cumulated the number of epitopes containing the same amino acid site in each protein. The numbers at each amino acid site were linked together to form a broken line chart for each protein. From each structural protein, two conserved peptide sequences that could potentially provide the largest number of epitopes were selected for the chemical synthesis (Sangon Biotech, Shanghai, China) (Figure 1b).

### 2.3. Peripheral Blood Mononuclear Cells (PBMCs) Isolation

Peripheral blood samples were collected. PBMCs were isolated within 4 h after collection using Ficoll-paque (Nycomed Pharma, Oslo, Norway) density-gradient centrifugation. Isolated PBMCs were cryopreserved in fetal bovine serum (FBS, Gibco, Grand Island, NY, USA) with 10% DMSO (Gibco) and stored in liquid nitrogen until use. Frozen PBMCs were thawed and rested in an incubator for 1 h before culture.

### 2.4. IFN-γ Enzyme-Linked Immunospot (ELISPOT) Assay

For analyzing the existence of peptide-specific memory T cells in unexposed healthy donors, IFN-γ ELISPOT assay was performed to assess the ex vivo response of PBMC to peptides, using ready-to-use human IFN-γ ELISPOT kit (Dakewe Biotech, Shenzhen, China). PBMCs were plated in triplicate with 2 × 10^5^ cells per well, with peptide added to ELIPSOT wells at 4 μM concentration. Plates were incubated overnight at 37 °C for 22 h. The following ELISPOT assay was performed according to the manufacturer’s instructions. The number of spot-forming units was determined on a C.T.L. ImmunoSpot S6 Analyzer and analyzed by ImmunoSpot v6.0 software (Cellular Technology Ltd., Cleveland, OH, USA).

### 2.5. Flow Cytometer Analysis and Sorting

Response of PBMCs to stimulation with conserved peptides in vitro was analyzed by flow cytometer. PBMCs were suspended at density of 2 × 10^6^/mL in a T cell medium consisting of RPMI-1640 (Gibco) supplemented with 5% human AB serum (Sigma, St. Louis, MO, USA). PBMCs were cultured in vitro with peptide at 4 μM concentration for different time according to the assays.

For peptide-specific naïve T cells analysis, PBMCs were plated in 24-well plate for culture of 72 h. When PBMC is stimulated with peptide in vitro, CD137 expression in T cells could lasted for a relatively longer time [30]. For the dynamic analysis of T cell activation, PBMCs were plated in 24-well plate and the culture time ranged from 0 days to 14 days with one sample corresponding to each day. IL-2 (50 IU/mL, PeproTech, Rocky Hill, NJ, USA) were first added on day 3, then half of the medium were changed every 2 days with IL-2 added to the T cells (50 IU/mL).

Cells were collected and incubated with cell surface antibody for 30 min on ice, and subjected to flow cytometry for analysis of the stimulation or the phenotype of T cells (Thermo ATTUNE NXT). DMSO without peptide was used in control group. The following are the antibodies used from Biolegend (San Diego, CA, USA): FITC-CD3 (300306), PE-CD4 (300507), APC-CD8 (301014), 7-AAD (420403), BV421-CD137 (309820), APC/Cy7-CD45RA (304127), and BV605-CCR7 (353223). The results were analyzed off-line with FlowJo software. Ratio of activated T cell proportions more than 1.5 between the experimental and the control groups was considered positive.

For sorting peptide-specific T cells, PBMCs were cultured with peptide in 6-well plate in vitro for 2 weeks as the first-round stimulation, with medium changed as described above. Then, the rested cells were re-stimulated by co-culture with peptide-loaded autologous PBMCs for 24 h. The target cells were set as 7-AAD^–^CD3^+^CD8^+^CD137^high^ and sorted in BD LSRFORTESSA X-20. The sorted cells were subjected to TCR β repertoire sequencing.

### 2.6. TCR β Repertoire Sequencing

TCR β libraries were prepared from isolated RNA using the ImmuHub TCR profiling system (ImmuQuad Biotech, Hangzhou, China). Briefly, a 5′ rapid amplification of cDNA ends (RACE) unbiased cDNA amplification protocol was used, with unique molecular identifiers (UMIs) introduced in cDNA synthesis to control bottlenecks and to eliminate errors in PCR and sequencing segments. Sequencing was performed on an Illumina NovaSeq system in PE150 mode.

Map V, D, J and C segments with the international immunogenetics (IMGT) to extract CDR3 regions and assemble the clonotypes. Determined the resulting nucleotide and amino acid sequences of CDR3 clone and discarded the out-of-frame and stop codon sequences from the TCR β repertoire. TCR β clones sharing the same nucleotide sequence of CDR3 were defined as the same TCR β clonotype.

### 2.7. Statistics

Data were reported as the median ± standard deviation. Student’s *t*-test was used to compare two groups, and one-way analysis of variance (ANOVA) was used to compare three or more groups. All results were plotted in Prism 8 (GraphPad Software Inc., San Diego, CA, USA).

## 3. Results

### 3.1. Conserved Antigens from the Structural Proteins

The SARS-CoV-2 structural proteins, including S, E, M, N, can be expressed in larger amounts than the non-structural proteins in host cells, therefore are advantageous in providing more antigens for processing and presentation for T cell immune response. We chose them as the targets for the selection of conserved antigens. To identify the potential epitopes that are conserved in the structural proteins of SARS-CoV-2, the protein sequences of 65 SARS-like viruses, including SARS-CoV and SARS-CoV-2, were aligned by Clustal Omega (v.1.2.4) algorithm (Table S1). Meanwhile, NetMHCpan4.0 was employed to predict the distribution of MHC-I epitopes in structural proteins of SARS-CoV-2 standard strain (Wuhan-Hu-1/2019). We selected the Oriental and the Caucasian populations, which have different HLA compositions, and checked whether there were differences in potential epitopes between the two populations (Supplementary Table S2).

The prediction results were weighted for HLA allele positive ratios and presented in terms of the cumulative number of potential epitopes in each amino acid site (Figure 1a). The two populations had similar cumulative epitope number curves, which seems to reflect, in part, similar immunogenicity of the viral protein between these two populations. Moreover, all the regions containing the largest number of potential epitopes in the four structural proteins were located within the conserved sequences (shaded segment), which indicated that these sequences might be applicable to a wider range of populations. To produce peptide antigens, the top two conserved sequences that contain the largest number of potential epitopes were selected from each of the four structural proteins for chemical synthesis, which resulted in a list of peptides including S15, S16, E2, E3, M1, M2, N16 and N18, except for E2 and E3 that were hard to be synthesized due to high hydrophobicity. S16 peptide was further divided into three overlapping segments for synthesis (Figure 1b). All the 8 synthetic peptides constituted the peptide pool. These peptides avoided the defining nonsynonymous mutations of variants reported so far, except for the S15 that covered the S_F888L substitution present in the variant of 20A/S:484K (B.1.525) (Supplementary Table S3). Though there were plenty of other mutations beyond the defining nonsynonymous mutations, most of them rarely persisted through the second transmission [31].

### 3.2. Conserved Antigen-Specific T Cell Responses

**Figure 2:**
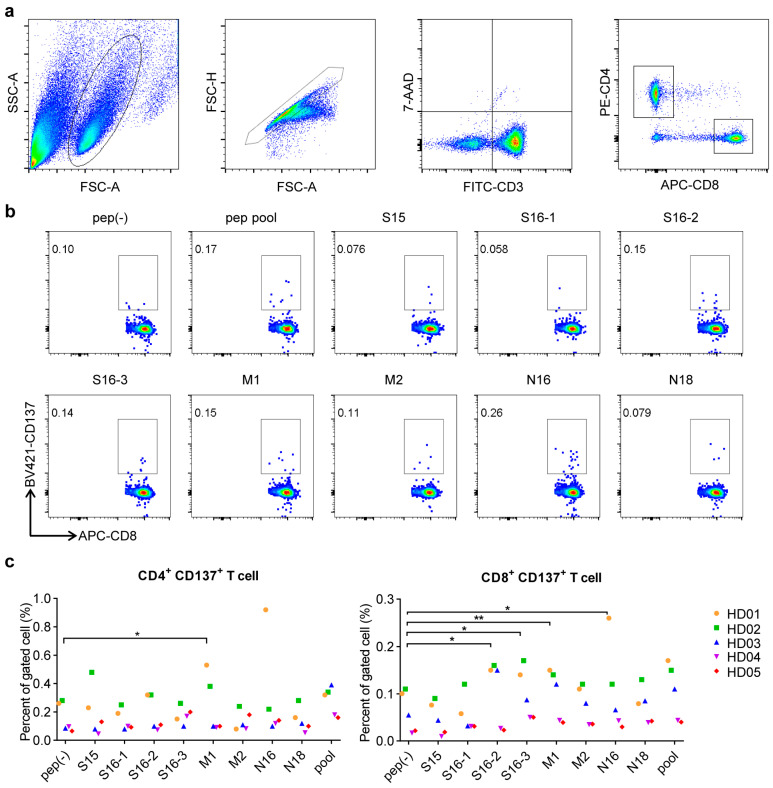
T cell immune responses to SARS-CoV-2 conserved antigens. Peripheral blood mononuclear cells (PBMCs) of 5 unexposed healthy donors were cultured with peptide pool for stimulation in vitro for 72 h, the T cell responses were assayed by flow cytometer. (**a**) Flow cytometry gating strategy. Only single, live, and CD8 or CD4 sole-expressing T cells were Boolean gated and used for analysis. (**b**) Responses of CD8^+^ T cell from unexposed healthy donor 01 (HD01) to each peptide were assayed. (**c**) Statistical graph of the responses of CD4^+^ and CD8^+^ T cell from all the 5 unexposed healthy donors. Paired *t*-test was used. (* *p* < 0.05, ** *p* < 0.01).

**Figure 3:**
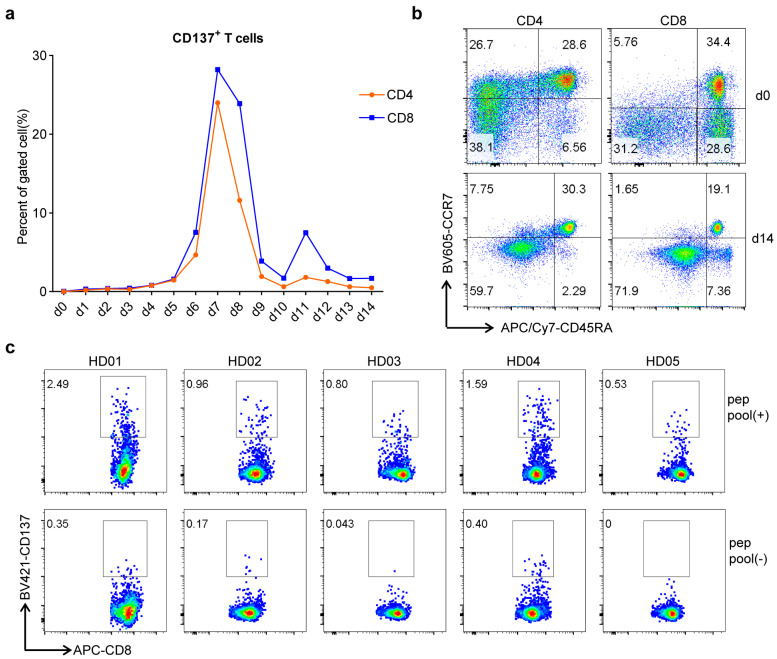
Fluorescence Activating Cell Sorter (FACS) of conserved antigen-specific CD8^+^ T cells from unexposed healthy donors. PBMCs of unexposed healthy donors were stimulated by culture with peptide pool in vitro for 2 weeks, for amplification of the peptide-specific T cells. (**a**) PBMCs from HD01 were cultured with peptide pool for stimulation with time range from 0 day to 14 days, for dynamic assay of T cell activation during the two-weeks culture. Each sample was collected every day for T cell activation analysis. (**b**) HD01 samples of d0 and d14, corresponding to pre- and post-stimulation, were assayed for T cell phenotype by flow cytometer using CD45RA and CCR7 biomarkers. (**c**) Cells were re-stimulated by co-culture with peptide pool loaded PBMCs overnight, and CD3^+^CD8^+^CD137^high^ T cells were sorted as conserved antigen-specific CD8^+^ T cells by FACS.

PBMCs of SARS-CoV-2 unexposed healthy donors (HD) were stimulated ex vivo with the peptide pool. However, almost no response was detected in IFN-γ ELISPOT assay, which reflected the almost non-presence of specific memory T cells for these peptides in unexposed healthy people (Supplementary Figure S1). Subsequently, we checked the existence of peptide-specific naïve T cells. PBMCs of HD were stimulated with peptide pool in vitro for 72 h. Flow cytometry assay showed that T cells were significantly activated (Figure 2). Although the proportions of activated CD4^+^ T cells were higher than those of CD8^+^ T cells, peptides had more obvious stimulatory effects on CD8^+^ T cells, which included S16-2, S16-3, M1 and N16 (Figure 2c). The stimulated PBMCs from HD01 were cultured in vitro for 2 weeks with T cells analyzed every day. Results showed that the proportion of activated T cells, including both CD4^+^ T cells and CD8^+^ T cells, increased slowly in the early stage, which reached a peak in day 7 (d7) and then began to decline till to the lowest level at day 14 (d14) (Figure 3a). After two-week stimulation, naïve T cells declined along with an increase of effector-memory T cells for both CD4^+^ and CD8^+^ T cell populations (Figure 3b). Accordingly, the proportions of conserved antigen-specific T cells increased, both of CD4^+^ and CD8^+^ T cells (Supplementary Figure S2). The different results from ex vivo and in vitro stimulations indicated that the peptide-specific T cells might be mainly derived from the naïve T cells in PBMCs, but it was also possible from rare, pre-primed T cells.

### 3.3. Characteristics of Conserved Epitope-Specific CD8^+^ TCR Repertoire

**Figure 4:**
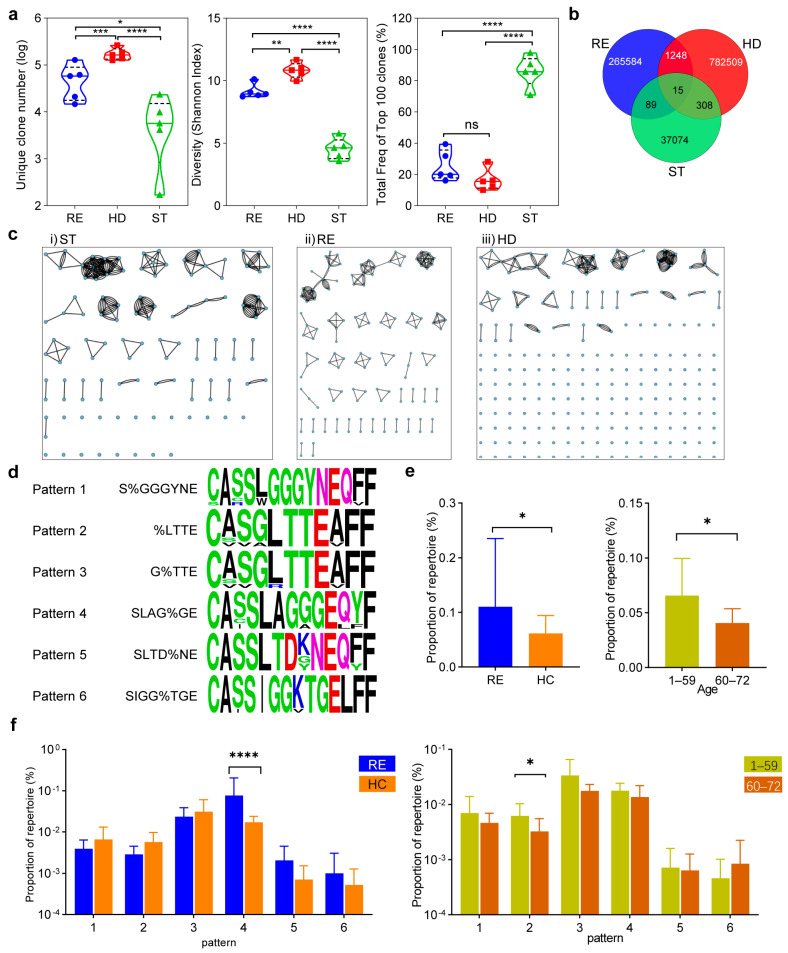
Conserved antigen-specific CD8^+^ TCR repertoire analysis. The sorted CD8^+^ T cells (ST), PBMCs of unexposed healthy donors and SARS-CoV-2 recovered patients (RE) were subjected to TCR β repertoire sequencing. (**a**) Comparison of overall TCR β repertoire diversity and clonality indices among the three groups. The full line is the median and the dotted-line is the quartile. (**b**) Amount of overlap for all unique clones and the public clones among the three groups. (**c**) TCR clones clustering of 3 groups by GLIPH2 algorithm. The dot represents a clone, and two dots are connected if they share a significant motif or have a similar CDR3 region. (**d**) Representative amino acid sequence patterns and logos of 6 prevalent CDR3 clusters in ST group. Clones in the same pattern possess a global similarity with only one amino acid variation in the CDR3 region. (**e**,**f**) Comparison of proportions of CDR3 cluster patterns between RE group and healthy cohort (HC), and between two age groups in HC. Analysis of the cumulative proportions of the 6 CDR3 cluster patterns (**e**), and the proportions of each CDR3 cluster pattern (**f**). Student’s *t* test (paired and unpaired) was carried out for two groups statistical analysis, and by ANOVA when more groups were analyzed (* *p* < 0.05, ** *p* < 0.005, *** *p* < 0.001, **** *p* < 0.0001, ns not significant).

The stimulated cells were co-cultured with peptide pool-loaded PBMCs overnight for re-stimulation, after which the CD8^+^CD137^high^ cells were sorted by flow cytometry (Figure 3c). The sorted T cell samples (ST) and their corresponding PBMCs (HD) were then subjected to TCR β repertoire sequencing, together with the PBMCs from 5 recovered volunteers (RE) of COVID-19. The three groups showed different TCR β clonal characteristics. The ST group possessed the smallest unique clone number and clone diversity but the highest cumulative proportion of top 100 clones, indicating the existence of dominant TCR clones. As expected, the number and diversity of TCR clones in RE group were significantly lower than those of HD group, which resembled the increased clonality of peripheral TCR repertoires of patients after viral infection (Figure 4a) [32]. This reflected that the immune system has not recovered to normal post-SARS-CoV-2 infection.

Although there were identical TCR clones across the three groups, they made up only a small minority (Figure 4b). However, in addition to identical TCR clones, different clones with similar CDR3 sequences may also possess the same epitope specificity [33]. The top 200 clones of each repertoire were collected and analyzed by GLIPH2 for clone clustering. TCRs of ST group were well connected and clustered, thereby illustrating the high sequence similarity among these clones (Figure 4c(i)), suggesting the existence of clones potentially recognizing same SARS-CoV-2 epitopes. The RE group showed the medium clustering characteristic (Figure 4c(ii)), while most clones of HD group were not clustered well (Figure 4c(iii)). According to the clustering results of the ST group, 6 globally similar CDR3 patterns, representing for the majority of the 5 donors with the largest number of clones (Figure 4d), were selected for clonal analysis in a TCR repertoire database. This cohort included 61 individuals of healthy cohort (HC) with age ranging from 3 to 72, whose TCR β repertoires were sequenced before the outbreak of COVID-19 (Supplementary Table S4). As a result, the RE group had TCR clones corresponding to 6 patterns, as a whole, higher than those in the HC group, which reflected the amplification of viral-specific T cell clones after infection; while within the HC group, TCR clones of the 6 patterns were more prevalent in the younger population, which could be ascribed to decreased repertoire diversity with age (Figure 4e). For the proportion of each pattern, TCR clone proportions of individual pattern showed no significant difference between RE and HC group, except for pattern 2. Similarly, only proportion of pattern 2 showed significant difference between populations of 1–59 and 60–72 years old (Figure 4f), suggesting that the number of TCR clones recognizing the conserved epitopes of SARS-like viral structural proteins was affected by exposure experience and age but to a lesser extent.

## 4. Discussion

It is now commonly speculated that the cross-reactive memory CD4^+^ T cells might be pre-existing against human “common cold” coronaviruses (hCoVs) including HCoV-OC43, HCoV-HKU1, HCoV-NL63, and HCoV-E [24]. Although the homology between hCoVs and SARS-CoV-2 is relatively low, it could meet the requirement of cross-reaction for CD4^+^ T cells, but more difficult for CD8^+^ T cells, which allow only one or two substitutions in epitopes [34]. This depends on the sequence homology of the selected epitope between hCoVs and SARS-CoV-2. Recent study has proved that CD8^+^ T cell cross-reaction recognizing homologous epitopes is also present in the unexposed populations [28]. However, there was no reaction with the peptide A11-P08, which was the only conserved peptide in the study. At present, some studies have proved the existence of SARS-CoV-2-specific CD8^+^ T cells, but there are few direct studies on viral conserved sequence-specific CD8^+^ T cells, which is one of the prerequisites of broad-spectrum CD8^+^ T cell immunity [23,35]. Unlike these studies, we specifically selected peptides conserved across different SARS-like viruses including SARS-CoV-2 and explored the presence of the corresponding specific CD8^+^ T cells in the unexposed population. This helps answer the question of whether there is a broad-spectrum CD8^+^ T cell immunity in the unexposed population, which may target different SARS-CoV-2 variants. Our results showed that unexposed healthy people do not have cross-reactive memory T cells recognizing the conserved antigens, which was possibly due to the very low homology of conserved antigens between hCoVs and SARS-CoV-2, suggesting that previous hCoVs infection and immunity can hardly preserve the specific T cells that recognize conserved SARS-CoV-2 antigens. This is in line with that the specific CD8^+^ TCR repertoire is present mainly in naïve T cells for the lack of prior stimulation in the unexposed healthy population. These results indicate that the conserved antigen-specific TCR repertoire is widely present in the population, though the TCR clone number and repertoire diversity decrease with age.

Non-Structural Proteins (NSPs) were not included in this study, which has been shown to have CD8^+^ T cell immunogenicity in other studies [22,24]. It is theoretically possible to speculate that CD8^+^ TCR repertoire recognizing the conserved antigens of NSPs might also exist in unexposed healthy population. Thus, the range of conserved antigens available for broad-spectrum immunity is predictably wider. Compared to subunit or mRNA vaccines targeting the S protein that is critical for SARS-CoV-2 infection and pathogenicity [36,37,38], the inactivated or live attenuated SARS-CoV-2 vaccines contain more conserved peptides of SARS-like viruses that would help stimulate broad-spectrum T cell immunity, though further comparative studies are warranted.

The limitations of our study include the sample size and lack of vaccinated cases. We adopted a combined strategy of small-sample assay in the wet lab first that was followed by a large-sample bioinformatic analysis in the dry lab. In this way, we tested results obtained from a small sample in a larger cohort. This turned out to be helpful in answering the scientific question of this study, that is, whether the unexposed population has broad-spectrum CD8^+^ T-cell immunity against SARS-CoV-2 through recognizing the conserved antigens. Meanwhile, as this study was of exploratory nature, we set recovered group as a substitution. Given the severity and predictably long lasting of COVID-19 pandemic, any increase of protective immunity or even anti-infection immunity could have a substantial impact on the progress of this pandemic.

Coordinated SARS-CoV-2 adaptive immune response of two arms, including neutralizing antibody and T cell, were associated with milder disease, highlighting the importance of both CD4^+^ and CD8^+^ T cells in protective immunity in COVID-19 [39]. For the lack of pre-existing memory CD8^+^ T immunity in the unexposed population and the increasing virus variants, the broad-spectrum CD8^+^ T immunization strategy is worthy of further exploration.

## 5. Conclusions

Together, this study demonstrated that unexposed healthy individuals do have CD8^+^ TCR repertoire that could recognize conserved antigens of SARS-CoV-2 structural proteins, and the repertoire might be widespread in the unexposed healthy population. The TCR repertoire is one of the prerequisites of broad-spectrum CD8^+^ T cell immunity and is tolerant of the SARS-CoV-2 mutations.

## Data Availability

The data presented in this study are available in article.

## Supplementary Materials

The following are available online at https://www.mdpi.com/article/10.3390/vaccines9101093/s1, Figure S1: Representative assay of memory T cells in unexposed healthy donors. PBMCs from HD01 were stimulated with peptide for 22 h and subjected to IFN-γ ELISPOT assay, with triplicate wells for each peptide. DMSO without peptide was added to Pep (-) as the negative control. The ex vivo response of PBMCs represented the existence of memory T cells. On the left is the scan gram of each well in the ELISPOT plate, and the corresponding statistical graph of number of spot-forming units was on the right. The threshold for a positive response is set at a net 50 spots per 1 × 10^6^ cells. No immune response was detected. Figure S2: T cell immune responses to SARS-CoV-2 conserved antigens after two-week stimulation. PBMCs of HD01 were stimulated in vitro by peptide pool for 2 weeks, after that T cells were re-stimulated with each peptide to check the immune response. Table S1: SARS-like viruses and conserved peptides. Table S2: MHC-I epitopes prediction for the Oriental and Caucasian populations. Table S3: SARS-CoV-2 variants defining mutations. Table S4: CDR3 clone patterns in TCR β repertoires of healthy cohort. TCR β repertoire seq: TCR β repertoire sequencing data of the three groups including ST, RE, HD, with 5 samples for each group.
