# Supplementary figures and images for: Specific CD8+ TCR Repertoire Recognizing Conserved Antigens of SARS-CoV-2 in Unexposed Population: A Prerequisite for Broad-Spectrum CD8+ T Cell Immunity
Source: Vaccines (Basel). 2021 Sep 28;9(10):1093. doi: 10.3390/vaccines9101093 (PMC8541101; doi:10.3390/vaccines9101093)

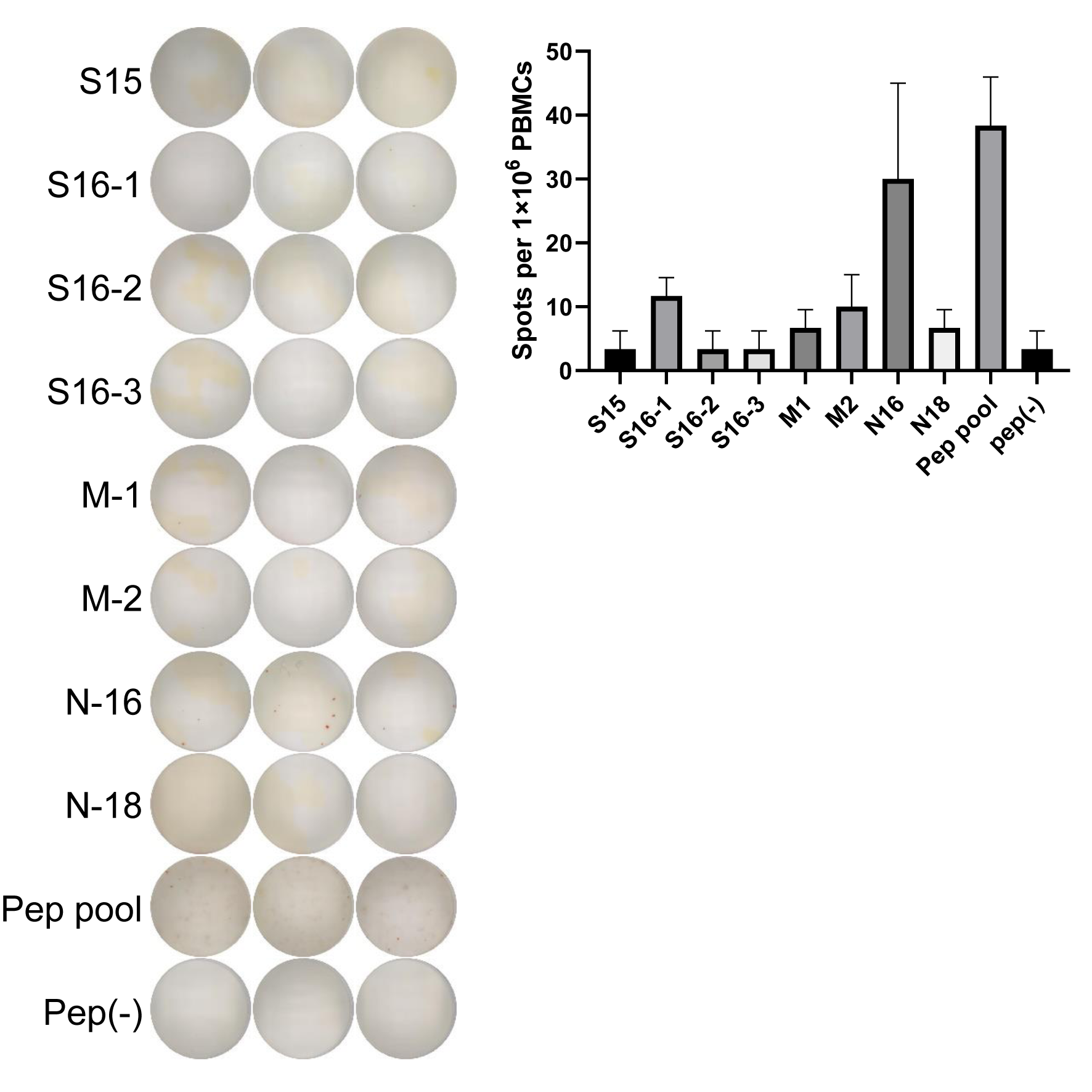

Supplement: Supplementary file 1 [file vaccines-09-01093-s001.zip › Fig S1.tif]

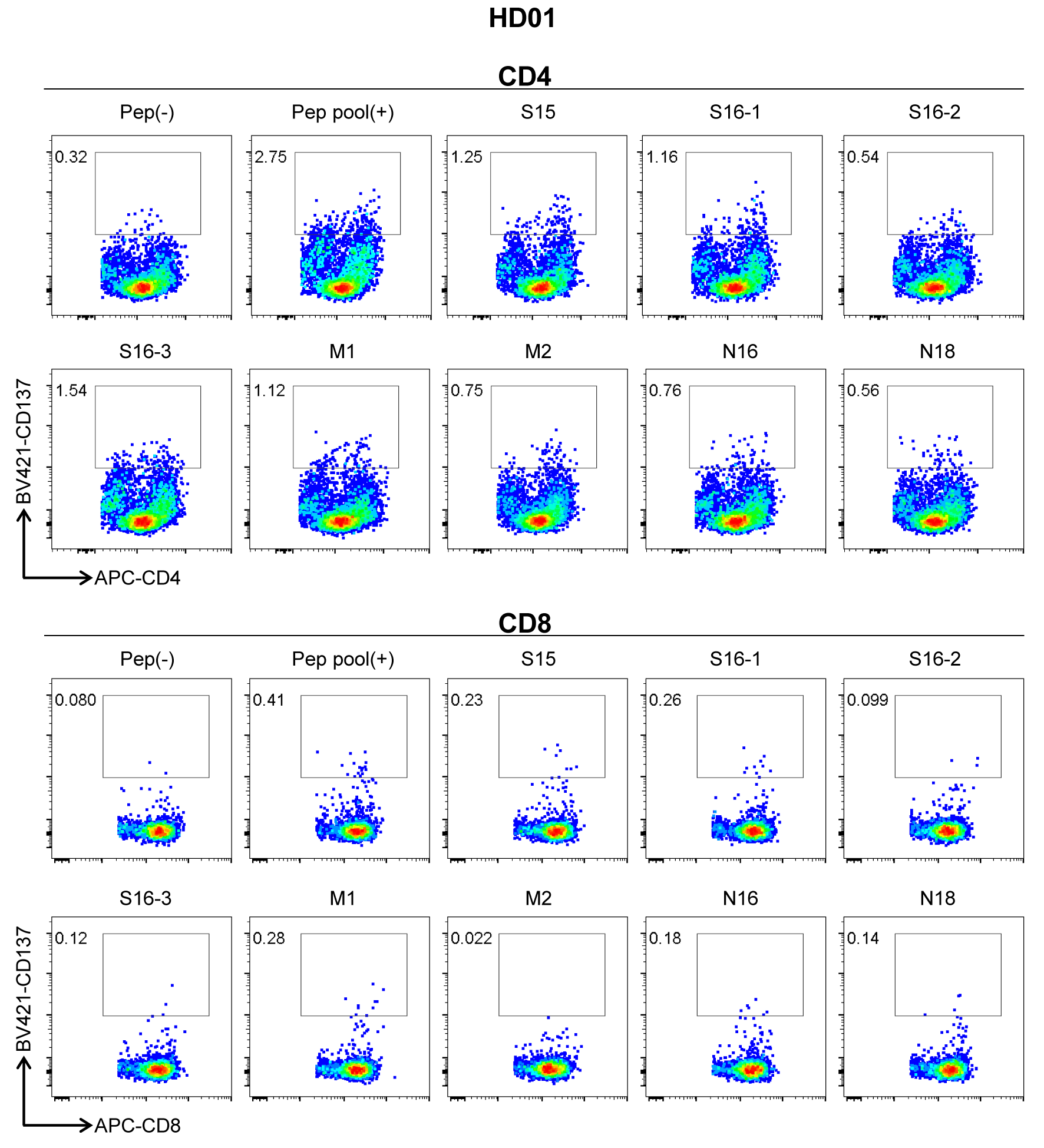

Supplement: Supplementary file 1 [file vaccines-09-01093-s001.zip › Fig S2.tif]
